# *Aspergillus* Sinusitis: Risk Factors and Phenotyping

**DOI:** 10.3390/jcm13092579

**Published:** 2024-04-27

**Authors:** Lena Hafrén, Riitta Saarinen, Rane Kurimo, Milla Viljanen, Marie Lundberg

**Affiliations:** Department of Otorhinolaryngology-Head and Neck Surgery, HUS Helsinki University Hospital, University of Helsinki, 00029 Helsinki, Finland

**Keywords:** chronic rhinosinusitis, etiology, fungal disease, fungal ball, *Aspergillus*

## Abstract

**Background**: *Aspergillus* can cause fungal rhinosinusitis (FRS). We aimed to identify risk factors for sinonasal *Aspergillus* disease. **Methods:** Patients with a positive sinonasal mycological culture for *Aspergillus* species diagnosed in our hospital located in a continental climate were included in the 9-year retrospective study. **Results:** Of the 86 patients, 3 had invasive FRS (IFRS), 51 had fungal ball (FB) disease, and 32 had chronic rhinosinusitis with fungus (CFRS). In the IFRS group, all patients had a malignancy and were immunocompromised. Allergies, allergic rhinitis, asthma, nasal polyps, and the use of inhaled and nasal steroids were more common in the CFRS group, and IgE levels were greater than those in the FB and IRFS groups (*p* < 0.05). **Conclusion:** FB disease is a relatively symptom-free single-sinus disease among elderly individuals, and IFRS is dominant among immunocompromised patients. We discovered a third patient group, predominantly with nasal polyps, atopy, asthma, and elevated blood IgE and eosinophils, that did not fulfill the allergic FRS (AFRS) criteria. It is possible that a less fulminant category of underdiagnosed AFRS exists in cold climates. Treatment with local debridement is usually sufficient for FRS, apart from IFRS, and relapses are not common in cold climates.

## 1. Introduction

Fungi are ubiquitous in our environment, with *Aspergillus* spp. being among the most common. Fungi are also found on the mucosa of almost all healthy individuals. In some cases, fungi can cause nasal sinus disease, but the definitions and categorizations are not clearly defined [1,2]. In immunocompetent patients, the most common type of fungal disease is a noninvasive ball of fungal hyphae. Fungal balls (FBs) usually arise in a single maxillary or sphenoid sinus, and the inflammatory process resembles that of a foreign body reaction involving the accumulation of casein-like dense material. There is a female predisposition, and it typically occurs late in adulthood. FB disease can be indolent for a long period, but most patients are symptomatic upon diagnosis [3,4]. The incidence of this disease is increasing [5], possibly because of the aging population or improved diagnostic methods, but other explanations are possible as the etiology is largely unknown. Prior endodontic treatment and ostial closure are known risk factors [6]. Primary treatment involves surgical removal, but chronic inflammation can occur, and the recurrence rate is 4–18% [7].

Especially in humid and warm climates, fungi can cause noninvasive, allergic fungal rhinosinusitis (AFRS) that resembles chronic rhinosinusitis with nasal polyps (CRSwNP). The diagnostic criteria include nasal polyposis, fungi on staining, eosinophilic mucin, type I hypersensitivity to fungi, and characteristic, usually unilateral findings in computed tomography (CT) [1,8,9]. The minor criteria for AFRS include asthma, fungal culture, bone erosion on CT, eosinophilia, and Charcot–Leyden crystals in mucin.

In immunocompromised patients, fungi can cause invasive or even fatal disease [7]. The most common underlying predisposing factors are hematologic malignancies and uncontrolled diabetes, followed by HIV/AIDS, malnutrition, transplant-related immunodeficiency, and neutropenia. Fungi invade and infiltrate neurovascular structures, resulting in tissue infarction, necrosis, and thrombosis [9,10]. Unilateral disease is here again typical, and treatment includes a combination of surgical debridement, systemic antifungal therapy, and a reduction in immunosuppression.

*Aspergillus* is one of the most common pathogens of fungal rhinosinusitis, and several *Aspergillus* species have been associated with this disease. Transformation from noninvasive to invasive disease has been suggested for immunocompromised patients, suggesting the need for active treatment. Because very few risk factors have been identified, especially for FB disease, we aimed to characterize patients with *Aspergillus* disease, investigate the underlying etiological factors, and compare the outcomes of different cases of *Aspergillus* sinusitis.

## 2. Materials and Methods

We included all patients with a positive nasal sinus mycological culture for any *Aspergillus* species diagnosed at Helsinki University Hospital (HUS), a tertiary-care referral center, between January 2010 and December 2018. We retrieved patient information retrospectively from the HUS laboratory database and manually collected patient data, including age, sex, weight, height, blood eosinophil and immunoglobulin E levels, smoking history, symptoms, infected sinuses, exact location of the nasal sinus sample, *Aspergillus* species, smear sample results, and bacterial sample results. In addition, we collected data on the preceding year’s antibiotic treatments, cancer therapy, peroral and local steroid use, medications affecting immunity, and information on allergies, nonsteroidal anti-inflammatory drug (NSAID) intolerance, head and neck surgeries, and comorbidities, including autoimmune diseases and past or present malignancies. We calculated body mass index (BMI) and assessed the Lund–Mackay (LM) score from CT scans. The LM score describes sinus opacification and obstruction of the osteomeatal complex on a scale from 0 to 24 [11]. Follow-up data on recovery, symptoms, and mortality were also collected for a minimum of two years.

The mycological samples were nonsterile. They were cultivated and morphologically analyzed at the HUS microbiology laboratory. Susceptibility testing was performed if requested by the clinician. In addition, a smear sample was prepared for each case. We recorded the exact location of the nasal sinus sample; mycological, bacterial, and smear sample results; and exact *Aspergillus* species.

We analyzed clinical and demographic data using descriptive statistics and IBM^®^ SPSS^®^ Statistics, version 25. We used Fisher’s exact test or the chi-square test for categorical variables, the *t* test for comparisons of means, and the Mann-Whitney U test for nonparametric continuous variables. Spearman’s correlation was used to assess associations between variables. We considered *p* ≤ 0.05 to indicate statistical significance.

## 3. Results

### 3.1. Phenotypes

**Figure 1:**
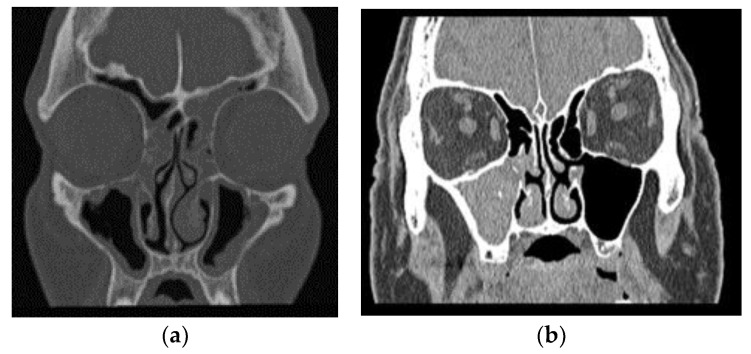
Typical CT scan of a patient with (**a**) chronic rhinosinusitis and (**b**) one sinus fungal ball.

We encountered 86 patients, 55 (64%) of whom were women. In a nine-year period, in our catchment area of 1.5 million persons, this gives an incidence of 6.4 in 1 million per year. The mean age was 65.4 years (range: 29.5–90.3 years), with 64.1 years for females and 66.2 years for males. A total of 3 patients had IFRS, and 51 patients had a classical single-sinus FB or, in some cases, local spread into adjacent sinuses. The remaining 32 patients had a noninvasive disease, but radiological findings of chronic sinusitis in numerous sinuses were mostly bilateral (Figure 1). These patients came from a dry and cold climate where AFRS is usually not observed. Fungal hypersensitivity was not tested, eosinophilic mucin was not found on staining, and CT findings were not typical; thus, the AFRS diagnostic criteria were not fulfilled [8]. In this paper, we refer to this patient group as having chronic fungal rhinosinusitis (CFRS).

### 3.2. Symptoms and Findings

Patient and disease characteristics are described in Table 1 and Table 2. The most common symptom overall was rhinorrhea (59%), followed by blockage of the nose (45%), pressure in the sinuses (41%), and facial pain (35%). Eighteen patients (21%) were symptom-free, and some reported symptoms only when they were asked. Rhinorrhea was more common in the CFRS group (*p* = 0.014). Pressure and rhinorrhea were strongly correlated with *Aspergillus* in the maxillary sinuses (r = 0.26, *p* = 0.01, r = 0.31, *p* = 0.004), and rhinorrhea was strongly correlated with bilateral *Aspergillus* (r = 0.25, *p* = 0.02). Pain was correlated with *Aspergillus* in the frontal sinuses (r = 0.24, *p* = 0.02). The disease was an incidental finding on a CT scan or a magnetic resonance image in 24 patients (28%).

Patients with IFRS (N = 3) were, on average, 64.1 years old, had a normal weight (BMI: 24.3 kg/m^2^), presented with malignancy (100%), and were receiving immunosuppressive medication (100%). Two of the three patients had leukemia with disseminated *Aspergillus* in the blood and lungs. Sinuses were suspected to be the origin of the disease, as no other focus was found, even though the sinonasal disease was minimal and indolent. The patients were treated with a combination of sinus lavage and antifungal therapy. The third patient was a smoker with type II diabetes who was receiving prednisolone treatment for polymyalgia rheumatica and ongoing treatment for prostate carcinoma. He had acute IFRS invading the orbital apex, resulting in blindness. Treatment consisted of radical surgery and antifungal therapy. No mortality from IFRS was observed in our cohort. The IFRS patients were not allergic or asthmatic, did not have a CRS diagnosis before IFRS, and consequently did not use inhaled or nasal corticosteroids during the preceding year.

The average age of the patients with FB disease (N = 51) was 69 years, which was significantly greater than that of patients in the other groups (*p* = 0.02). They were predominantly women (66.6%), as was the case for all FRS types. On average, their LM score was low (3.2), and the disease was bilateral in only three patients (5.9%). No sinuses other than the maxillary sinus (88.2%) or sphenoid sinus (17.5%) were affected. Although 37% were incidental findings, 31–53% of the patients suffered from rhinorrhea, nasal obstruction, pressure, and pain.

The patients with CFRS (N = 32) were significantly younger than those in the other groups, with an average age of 59.7 years compared to 69.1 and 64.1 years in the FB and IFRS groups, respectively (*p* = 0.02). They had a significantly higher LM score of 8.4 (*p*< 0.001), a greater proportion of the patients had a history of nasal polyposis (59.4%, *p* = 0.008), and 34% had bilateral *Aspergillus* findings. Unlike in FB disease, all paranasal sinuses were affected and symptomatic, with rhinorrhea (89%), nasal obstruction (68%), pressure (61%), and pain (50%). CRS diagnosis preceded *Aspergillus* culture in 66% of the patients, compared to 31% in the FB group and 0% in the IFRS group (*p* = 0.006). Previous sinonasal surgery was also more common (72%, *p* < 0.001) compared to 28% in the FB group.

### 3.3. Cultures

**Figure 2:**
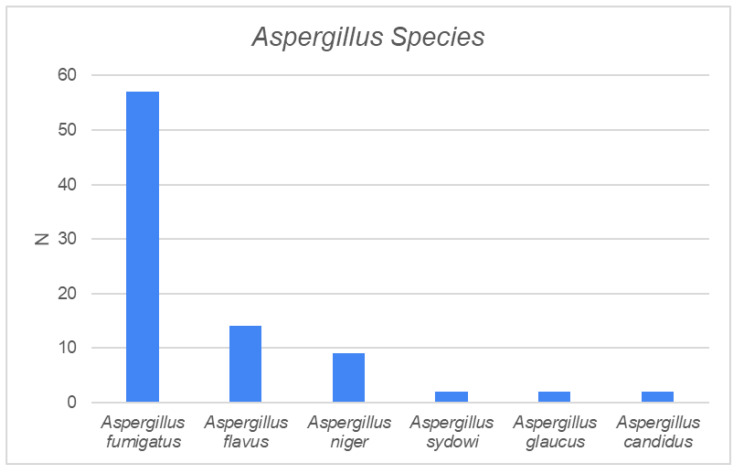
*Aspergillus* species found in mycological cultures from the paranasal sinuses.

**Figure 3:**
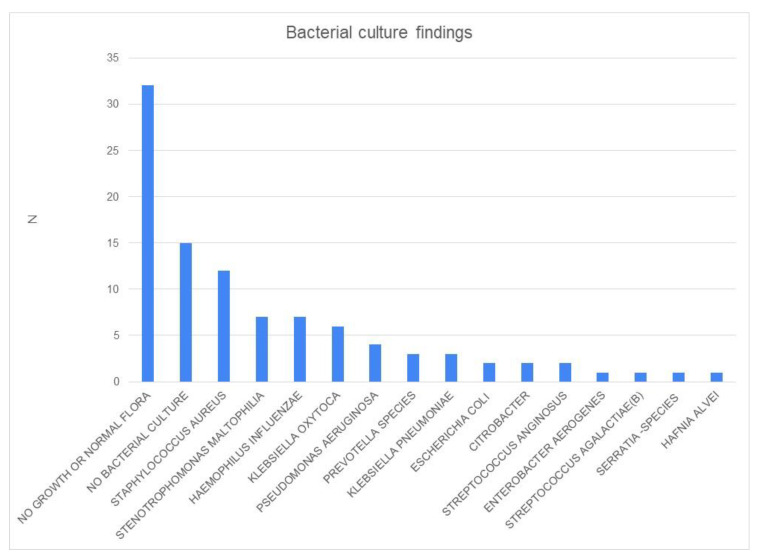
Bacterial culture results in the paranasal sinuses concomitant with *Aspergillus* findings.

The main *Aspergillus* species were *A. fumigatus* (66%), *A. flavus* (16%), and *A. niger* (12%) (Figure 2). *A. fumigatus* was the most common type among all three phenotypes. There was no significant difference in the culture findings among the three groups, although the rare *A. sydowi* was found only in the CFRS group (N = 2), and *A. glaucus* (N = 2) and *A. candidus* (N = 2) were found only in the FB group (Table 2). IFRS was caused by *A. fumigatus* in two patients and by *A. niger* in one patient. Bacterial cultures were obtained from 72 patients, and *Staphylococcus aureus* was the most common pathogen (Figure 3). Encountered bacterial pathogens were treated with a targeted antibiotic if the patient was symptomatic. There was no significant difference in the culture findings between the groups.

### 3.4. Treatment

**Figure 4:**
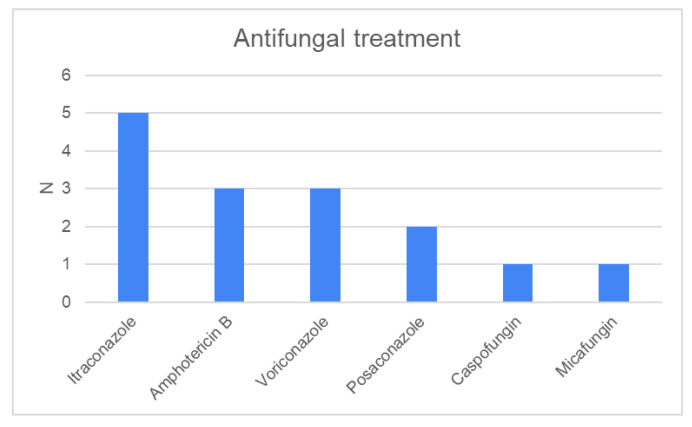
Systemic antifungal treatment for *Aspergillus* rhinosinusitis.

The most common treatment for *Aspergillus* sinusitis in our cohort was endoscopic endonasal sinus surgery (N = 61): middle meatal antrostomy (N = 47), inferior meatal antrostomy (N = 28), or both (N = 22), in combination with other procedures (Table 3). In the FB group, 78% of patients underwent surgery, and in the CFRS group, 61% of patients underwent surgery; however, in some patients, surgery preceded fungal positivity. More than half of the patients underwent sinus punctures or postoperative lavage. All patients with IFRS, 16% of patients with FB disease, and 9% of patients with CFRS were treated with one or several antifungal medications before and/or after surgical intervention (Figure 4). Eleven patients were lost to follow-up. At the three-month follow-up after the intervention, 77% (65/84) of the patients were considered cured of fungal disease. During the follow-up of two to ten years, only one patient with NSAID-exacerbated respiratory disease (NERD) relapsed with *Aspergillus* sinusitis.

### 3.5. Risk Factors

Compared with FB disease and CFRS, having a malignant disease or chemotherapy in the past year was a risk factor for IFRS (*p* = 0.018, *p* < 0.001, respectively). The amount of immunosuppressive oral medication taken in the past year was greater in the CFRS and IFRS groups than in the FB group (*p* < 0.001). In the CFRS group, the immunosuppressive medication used was corticosteroids. Allergies, allergic rhinitis, asthma, and nasal polyps were more common in the CFRS group than in the FB and IRFS groups (*p* = 0.03, *p* = 0.001, *p* = 0.001, and *p* = 0.008, respectively). The levels of blood eosinophils and IgE were greater in the CFRS group (*p* < 0.001, *p* = 0.04). This was also true for the use of inhaled steroid medication and nasal steroids (*p* = 0.006 and *p* = 0.001, respectively). More than half (N = 48, 57%) had at least one course of antibiotics for upper respiratory tract infections during the previous year preceding the diagnosis of *Aspergillus* sinusitis. This was especially true for the CFRS group, in which 69% of patients (N = 22) were treated with antibiotics, but there was no statistically significant difference between the groups. Smoking status, diabetes status, BMI, sex, and autoimmune disorders did not differ between the groups.

To study risk factors on a population basis, we compared the prevalence of certain diseases in the cohort’s phenotypes to the population mean of the corresponding age. We found that the prevalence of asthma and NERD was much greater in the CFRS group than in the population mean: 44% vs. 12% for asthma [12] and 19% vs. 0.8% for NERD [13] (*p* < 0.001 for both). The prevalence of nasal polyposis was greater in both the CFRS (59%) and FB (28%) groups compared to the population (4%) [14] (*p* < 0.001). A history of malignancies was much more common in all phenotypes: 26% in the FB, 25% in the CFRS, and 100% in the IFRS group, compared to 5% in the population of the same mean age [15] (*p* < 0.001). In the cohort, hematologic malignancies were overrepresented compared to the population (8% vs. 0.6%) [16], as was polymyalgia rheumatica (5% vs. 2%) [17] (*p* < 0.001 for both). The prevalence of smoking [18] and diabetes [12] was similar to that in the Finnish population, and the average BMI of our patients was normal.

## 4. Discussion

This study aimed to characterize the sinonasal disease caused by *Aspergillus* and analyze the risk factors for this disease. The diagnostic grouping of FRS has evolved recently, distinguishing invasive and noninvasive FRSs. The noninvasive FRS can be further divided into FB and AFRS subcategories [1,19]. However, we found that it is not easy to allocate patients to a correct group. The symptoms and findings sometimes overlap or contradict each other. To ascertain the specific subgroup, one needs to consider previous examinations, mycology, histopathology, immunology, hypersensitivity, and imaging.

In our CFRS group, certain characteristics of AFRS were present: 58% had allergies, 44% had asthma, 66% had unilateral CRS, and 59% were female. On the other hand, only 59% had polyposis, and the average age was much greater than that reported in AFRS. Only one patient relapsed, and 72% were symptom-free after three months—features that are uncommon for AFRS [9,20]. None of our patients fulfilled the AFRS diagnostic criteria [8], and the climate zone of our center is not typical for this disease. It is possible, however, that AFRS is not readily suspected and is thus less frequently diagnosed in colder climates and is more common than previously thought. A cold climate might also alter the AFRS phenotype to a less fulminant phenotype. It is also possible that some of the patients had a saprophytic *Aspergillus* infection, which often causes an infection in inflamed mucosa after surgical intervention [6]. However, 38% of the patients had not undergone any surgery, but all had chronic rhinosinusitis and mucosal inflammation. It has been speculated that saprophytic colonization could be the starting point of fungal ball development. To achieve a more specific classification, we suggest that hypersensitivity testing and eosinophilic mucin staining should be part of the diagnostic work-up, especially when treating *Aspergillus* sinusitis in patients with CRSwNP with atopy and asthma [20].

Fungal colonization can impair mucociliary clearance and thus facilitate the formation of polyps [21]. The association with nasal polyposis could be explained by the blockage of ostia, or nasal polyps could be a consequence of *Aspergillus*-associated CRS. It is also possible that the continuous use of local or inhaled corticosteroids creates a feasible environment for *Aspergillus* growth. Here, we observed that asthma, allergic rhinitis, nasal polyposis, and inhaled and nasal corticosteroids were associated with CFRS. A limitation of the retrospective setup is that we do not know if the corticosteroids and antibiotics that 50% of patients were prescribed within a year before diagnosis were prescribed due to symptoms caused by *Aspergillus* or if they were prescribed for other symptoms and thus acted as risk factors for the development of the disease. Additionally, polymyalgia rheumatica, treated with oral corticosteroids, was highly prevalent in this cohort, but the number of patients was only four, so no certain conclusions can be drawn. Other rheumatic diseases, however, were not overrepresented, whereas immunosuppressive medication was common in both the CFRS and IFRS groups.

Malignancies and immunosuppressive states are known to be associated with IFRS [1,8,9], and all patients with IFRS in this cohort had active malignancies. In addition, malignancies were more prevalent in the FB and CFRS groups than in the corresponding age-matched population, which, to our knowledge, has not been previously reported. These results need further confirmation in registry studies. HIV/AIDS is a known risk factor for FRS worldwide, but we had no cases, presumably because patients with HIV in Finland are generally immunocompetent due to free treatment and close, mandatory follow-up [22]. A similar explanation is possible for diabetes, which is a reported risk factor for IFRS and FB disease [23,24,25]. We could not confirm this, but early screening, good monitoring, and active, low-cost treatment of diabetes is standard in Finland.

In our study, the female sex was dominant (64%), which has been shown in previous studies [26]. The reason is unknown. It has been hypothesized that the longer life expectancy of women could explain this difference, but Kim et al. reported a female predominance in younger cohorts [24]. In our study, the mean age did not differ between men and women.

As in previous studies [27,28], treatment schemes were largely dependent on surgery, with subsequent debridement and lavage. All sinus *Aspergillus* infections were primary, none experienced a recurrence of *Aspergillus* growth, and 78% were symptom-free at the three-month follow-up. Only one patient with NERD experienced recurrence after six years, indicating that *Aspergillus* treatment was predominantly successful. Antifungal therapy was rarely needed if the patient was immunocompetent and there was no sign of invasive disease. None of our patients received long courses of postoperative oral corticosteroids, which are recommended by some authors for the treatment of AFRS in warm climates [29].

As expected, *Aspergillus* sinusitis was more common in the elderly population. The mean age was 63.5 years in our cohort, whereas the mean population age in Finland is 43.4 years [30]. In our study, older age was a risk factor, especially for FB disease, and consequently for *Aspergillus* infection in a single sinus. FB disease was the most common type of *Aspergillus* infection in our cohort.

IFRS is rare, and we had only three cases of *Aspergillus* IFRS during the 9-year follow-up period. Thus, it is difficult to draw conclusions. IFRS is a subtype of invasive aspergillosis (IA), which includes invasive disease in the lungs, central nervous system, or disseminated aspergillosis [31]. All our patients with invasive *Aspergillus* infection were immunocompromised, which is common in patients with IA, and were treated with long courses of antifungal therapy. In our cohort, IFRS did not result in death, although the literature reports a mortality of 20–80% [7]. This might reflect the fact that we only collected data from patients with *Aspergillus* FRS, and no patients with, e.g., *Mucormycosis* spp. were included [32]. It should be remembered that superficial FRS can develop into invasive and more severe disease, and at least in immunocompromised patients, all types of FRS should be treated in time [1]. We observed one patient with FB disease with slight local invasion of *Aspergillus* into the mucosa, which could have been the first stage of IFRS.

## 5. Conclusions

Categorizing *Aspergillus* sinusitis can be challenging. FB disease is a relatively symptom-free, indolent single-sinus disease among elderly individuals, and IFRS is dominant among immunocompromised patients. In our cohort, we observed a third patient group predominantly with nasal polyps, atopy, asthma, elevated blood IgE and eosinophil levels, and the use of local and oral corticosteroids that did not fulfill the AFRS criteria. It is possible that a less fulminant category of AFRS exists in cold climates.

Malignancies appeared to be a risk factor for all phenotypes compared to the average population. All types responded well to treatment, mostly surgical and/or local debridement, with additional antifungal therapy for IFRS. Relapses are uncommon in continental climates. The three most common species were *A. fumigatus*, *A. flavus*, and *A. niger.*

## Figures and Tables

**Table 1:** Patient demographics. FB = fungal ball. CFRS = chronic fungal rhinosinusitis. IFRS = invasive fungal sinusitis.

	Total N = 86		FB N = 51	CFRS N = 32	IFRS N = 3	
		Range	Mean	Mean	Mean	*p* Value
Age at diagnosis (years)	65.3	29.5–90.3	69.1	59.7	64.1	0.02 *
Body mass index (kg/m^2^)	25.7	18.8–39.8	26.2	25.1	24.3	0.55
Lund–MacKay Score	5.3	1–21	3.2	8.4	4.7	<0.001 *
Smoker’s pack years	6.4	0–70	4.7	9.0	10.0	0.42
Oral antibiotic courses in the past year	1.6	0–13	1.0	2.5	1.7	0.006 *
Blood IgE (kU/L) ^†^	19.5	3–699	36.6	195.1	N/A	0.04 *
Blood eosinophils (109/L) ^‡^	0.04	0–0.95	0.12	0.38	0.12	<0.001 *

^*^ *p* < 0.05. ^†^ Normal level: 0–110 kU/L. ^‡^ Normal level: 0.1–0.4 10^9^/L.

**Table 2:** Patient demographics. FB = fungal ball. CFRS = chronic fungal rhinosinusitis. IFRS = invasive fungal sinusitis.

	Total N = 86		FB N = 51		CFRS N = 32		IFRS N = 3		
	N	%	N	%	N	%	N	%	*p* Value
Male sex	31	36.0	17	33.3	13	40.6	1	33.3	0.793
Current smoker	10	13.2	3	6.7	6	21.4	1	33.3	0.111
Asthma	19	22.1	5	9.8	14	43.8	0	0.0	0.001 *
Any allergy	34	41.5	16	33.3	18	58.1	0	0.0	0.031 *
Allergic rhinitis	19	23.2	5	11.1	14	50.0	0	0.0	0.001 *
Nasal polyps	33	38.4	14	27.5	19	59.4	0	0.0	0.008 *
CRS diagnosis before fungal sinusitis	37	43.0	16	31.4	21	65.6	0	0.0	0.006 *
Past nasal or sinonasal surgery	38	44.2	14	27.5	23	71.9	1	33.3	<0.001 *
Any malignancy	24	27.9	13	25.5	8	25.0	3	100.0	0.018 *
Diabetes	15	17.4	8	15.7	6	18.8	1	33.3	0.717
Autoimmune disorder (other than asthma)	16	18.6	9	17.6	6	18.8	1	33.3	0.794
Chemotherapy during the past year	7	8.1	5	9.8	0	0.0	2	66.7	<0.001 *
Immunosuppressive oral medication	28	32.6	9	17.6	16	50.0	3	100.0	<0.001 *
Inhaled steroid medication	19	22.1	6	11.8	13	40.6	0	0.0	0.006 *
Nasal steroid medication	39	45.9	16	32.0	23	71.9	0	0.0	0.001 *
Antibiotic treatments in the preceding year	48	57.1	25	49.0	22	68.8	1	33.3	0.205
Affected sinus									
Frontal sinus	5	5.8	0	0.0	5	15.6	0	0.0	0.011 *
Sphenoid sinus	16	18.6	9	17.6	6	18.8	1	33.3	0.794
Maxillary sinus	72	83.7	45	88.2	25	78.1	2	66.7	0.343
Ethmoid sinuses	5	5.8	0	0.0	4	12.5	1	33.3	0.007 *
Bilateral *Aspergillus* findings	14	16.3	3	5.9	11	34.4	0	0.0	0.002 *
Incidental finding	24	27.9	19	37.3	5	15.6	0	0.0	0.058
Rhinorrhea	50	58.1	24	53.3	25	89.3	1	33.3	0.014 *
Nasal obstruction	39	45.3	20	44.4	19	67.9	0	0.0	0.055
Pressure on sinuses	36	41.9	18	40.0	17	60.7	1	33.3	0.264
Pain	29	33.7	14	31.1	14	50.0	1	33.3	0.311
Surgical treatment	59	68.6	40	78.4	19	61.3	1	33.3	0.008 *
Treatment with sinus lavage	45	52.3	22	43.1	22	68.8	1	33.3	0.060
Treatment with antifungal medication	14	16.3	8	15.7	3	9.4	3	100.0	<0.001 *
Symptom-free at 3 months	65	77.4	40	81.6	23	71.9	2	66.7	0.533
*Aspergillus* species									0.391
*fumigatus*	57	66.3	34	66.7	21	65.6	2	66.7	
*flavus*	14	16.3	10	19.6	4	12.5	0	0.0	
*niger*	9	10.5	3	5.9	5	15.6	1	33.3	
*sydowi*	2	2.3	0	0.0	2	6.3	0	0.0	
*glaucus*	2	2.3	2	3.9	0	0.0	0	0.0	
*candidus*	2	2.3	2	3.9	0	0.0	0	0.0	

* *p* < 0.05.

**Table 3:** Treatment for fungal sinusitis. The same patient could undergo a combination of the following surgeries and treatments.

	N
Medial meatal antrostomy	47
Inferior meatal antrostomy	28
Only medial or inferior meatal antrostomy	22
Only sinus punctures or irrigation at an outpatient clinic	17
Sphenotomy	10
Anterior ethmoidectomy	7
Turbinoplasty	4
Only nasal endoscopy with removal of fungus	4
Only local treatment (nasal steroid and nasal irrigation at home)	4
Endoscopic frontal sinus surgery	2
Septoplasty	2
Posterior ethmoidectomy	2
Polypectomy without opening of sinuses	2

## Data Availability

Data are available upon request from the corresponding author.
